# Comparative transcriptome analysis of silkworm, *Bombyx mori* colleterial gland suggests their functional role in mucous secretion

**DOI:** 10.1371/journal.pone.0198077

**Published:** 2018-05-31

**Authors:** Liangli Yang, Qiuping Gao, Junjun Dai, Guozhen Yuan, Lei Wang, Cen Qian, Baojian Zhu, Chaoliang Liu, Guoqing Wei

**Affiliations:** 1 College of Life Sciences, Anhui Agricultural University, Hefei, P.R.China; 2 Sericultural Research Institute, Anhui Academy of Agricultural Sciences, Hefei, P.R. China; Institute of Plant Physiology and Ecology Shanghai Institutes for Biological Sciences, CHINA

## Abstract

Colleterial glands (CG) present in the body of adult female of *Bombyx mori*, which can help adhere eggs on the surface of the host plants. Although this organ has been known for centuries, only morphology and its secretions have been studied. Their gene expression profiles and physiological roles remain largely unknown. Aided by high-throughput next generation sequencing (NGS), we reported the comparative transcriptome analysis of CG isolated from the H9 and the P50 strains of *Bombyx mori*. A total of 19,896,957 and 20,446,366 clean reads were obtained from CG of H9 and the P50 strains, respectively; then differential expression analysis was performed, and 1,509 differentially expressed genes (DEGs) were identified. Among them, 1,001 genes are up-regulated and 508 genes are down-regulated in P50 individuals compared with H9 individuals. The enrichment of GO (Gene Ontology) and KEGG (Kyoto Encyclopedia of Genes and Genomes) of DEGs confirmed that many DEGs were associated with “Amino acid transport and metabolism”, “Nucleotide transport and metabolism”, and “Inorganic ion transport and metabolism”, 25 of the DEGs related to the “ECM-receptor interaction passway”, “sphingolipid metabolism passway”, and “amino sugar and nucleotide sugar metabolism passway” were potentially involved in the process of CG development and mucus secretion. According to these data, we hypothesized that CG play an important role in providing favorable physiological environment for the glue secretion formation. In addition, GO enrichment and differential expression analysis of the DEGs in the CG indicate that this gland may be involved in the transporting of small solutes such as sugars, ions, amino acids and nucleotide sugar to the CG. Our findings lay the foundation for further research on CG function.

## Introduction

The silkworm, *Bombyx mori* L. (Lepidoptera: *Bombycidae*) has been domesticated for more than 5000 years for silk production [1–3]. In many Asian countries such as China, India, and many developing countries, it plays an important role in the economic development. And with the development of biotechnology, silkworm has been used as an important recombinant protein bioreactor [4–7]. *Bombyx mori* is an economically important insect and also used as a model organism, it is very importance in silkworm breeding and silkworm eggs preservation. *Bombyx mori* has a large number of mutants, so far more than 400 mutants are identified, among of them 200 mutants have been positioned [8–10]. The loose eggs trait is controlled by *Ng* (no glue) gene. As a result of mutation CG secret less or sometime no glue like substance. In *Ng* gene mutation, the phenotype is natural loose eggs and the genetic traits is dominant inheritance compared with viscous eggs [11–13].

The colleterial glands (CG) are an accessory organ in the female silkworm's reproductive system, which develop slowly during prepupal period, while develop rapidly with reserving large amount of accumulation of secretions before the moth emergence [14, 15]. The secretion of CG exhibits strong adhesiveness; it is released on the surface of the eggs as the female moth oviposition and fixed the egg at oviposition sites, and so play a protective and compensatory nutrition role for the eggs [16–18]. A number of studies have made significant progress in understanding the morphology and the secretions of CG, adhesive strength of the glue substances, and the physical and chemical properties of glue like substances [15, 16, 19, 20]. For example, Nakamarak and Arisawa *et al*. reported the development of CG and the mechanism of protein synthesis in CG of female silkworm moth [20, 21]. Katsuiko *et al*. described the adhesive strength of the glue substances in the CG of the female moth [17]. Jin *et al*. studied the proteome of CG and its *Ng* mutant in *Bombyx mori*. They observed that 31 different proteins highly express in normal tissue of CG than that of the *Ng* mutant; further they noted that 17 proteins greatly express in the mutant tissue [15, 22, 23]. However, the mechanism of the specific protein synthesis in CG during a short period and the glue substance secreted from CG is not well understood.

In recent years, with the development of the high-throughput next generation sequencing (NGS), espescial the used of Illumina HiSeqTM2500 platforms, it provides a more favorable opportunity for scientific development, and great improved the efficiency and speed of gene discovery [24, 25]. Expressed sequence tags (ESTs) and microarray techniques have been used to search for differentially expressed genes (DEGs) [26, 27]. For example, Hu *et al*. based on comparative transcriptome analysis, obtained numerous differentially expressed genes (DEGs) in *Microtus fortis* following infection with Schistosoma japonicum [28]. Diao *et al*. revealed the recent horizontal transfer of DNA transposons between different mosquitoes by using Next-generation sequencing [29]. This technique is widely used in the silk gland and other tissues of *Bombyx mori*. For example, Yang *et al*. indicated that some function such as metabolism become reduced as the downregulated expression of some associated genes, DNA synthesis decreases and midguts harbor less microflora during the molting stage [30]. Cheng *et al*. found that 400 orthologous genes might have experienced or are experiencing positive selection according to transcriptome sequencing of the wild silkworm *Bombyx mandarina* silk gland [31]. Chang *et al*. detected 282 up-regulated genes in the anterior silk gland (ASG), when compared to other parts of the silk gland by using the RNA sequencing technology [32]. Qian *et al*. indicated that 241 genes are differentially expressed between the two libraries in the analysis of differentially expressed genes between fluoride-sensitive and fluoride-endurable individuals in midgut of silkworm [33]. And the technique is also used in *Antherea pernyi*, such as Xin *et al*. identified 528 DEGs by analyzed transcriptomes of pupae after stimulation with lead to understaning the antioxidant defense system of *A*. *pernyi* [34]. Here, the development and physiological function of CG are resolved by using the RNA sequencing technology (RNA-Seq).

To investigate the molecular mechanism about CG development and secretion, the CG isolated from H9 (*Ng* mutation/ lay loose eggs) and the P50 (normal strains/ lay viscous eggs) were used for RNA-seq in this study, the DGE analysis was performed between the CG of H9 and P50 strains and some DEGs were validated through qRT-PCR. Our results provide insights for understanding the molecular mechanism about CG development and secretion of *Bombyx mori*.

## Materials and methods

### Silkworm rearing and colleterial glands collection

*B*. *mori* (*Ng* mutation H9 and P50 strains provided by the Key Laboratory of Sericulture, Anhui Agricultural University, China) were reared in our laboratory. The first three instars larvae were reared with fresh mulberry leaves at the condition of 26±1°C, 75±5% relative humidity, and 12 hours day/night cycle [35]. The rearing temperature for the last two instars was reduced to 24±1°C; Remaining conditions were unchanged. CG from the virgin moths of H9 and P50 were isolated respectively in phosphate buffered saline (PBS, pH7.4). Thirty CG were mixed to minimize individual genetic differences. The collected CG were stored in 1.5 ml microtubes at -80°C until RNA extraction.

### RNA extraction

The CG were frozen in liquid nitrogen and pulverized, 100 mg of samples were added directly into an RNAase free microcentrifuge tube containing 1.0 ml of TRIzol Reagent (TaKaRa, Japan) for RNA extraction. The total RNA was extracted separately using TRIzol Reagent. Two RNA samples were quantified by NanoDrop 2000 spectrophotometer (Thermo Scientific, USA), and the quality assessment of protein contamination (A_260_/_A280_ ratios) and reagent contamination (A_260_/A_230_ ratios) was examined by spectrophotometry. Agligent 2100 was used to detect the strength of the 28S/18S rRNA band in the sample. The spectroscopic A_260_/A_280_ readings must be 1.8 to 2.0, and the A_260_/A_230_ readings must be higher than 1.5 (2:1) [33, 36].

### Library preparation and sequencing

Enrichment of mRNA, fragment interruption, cDNA synthesis, and addition of adapters, PCR amplification and RNA-Seq were performed by Beijing BioMarker Technologies (Beijing, China). Among them total RNA was extracted by using NEBNext Poly(A) mRNA Magnetic Isolation Module (NEB, USA) to purify mRNA. Later the cDNA library was constructed using the NEBNext mRNA Library Prep Master Mix Set for Illumina (NEB, USA) and NEBNext Multiplex Oligos for Illumina (NEB, USA). About the concentration of cDNA library and the Insert Size were tested by Qubit 2.0 and Agilent 2100 respectively. Suitable fragments were selected as templates and sequenced by synthesis on an Illumina HiSeqTM 2500 using paired-end technology.

### Raw data processing

Raw data (raw reads) were firstly processed using in-house Perl scripts. We used high-quality reads for sequence alignment with the designated reference genome. In order to obtain clean and high-quality reads (Clean Reads) for sequence assembly, the raw reads were filtered by removing adaptor sequences, Primer sequences, and low-quality sequences (reads with ambiguous bases ‘N’). And the reference genome and gene model annotation files were directly downloaded from the silkworm genome database (http://ftp.ensemblgenomes.org/pub/metazoa/release-24/fasta/bombyx_mori/). Through sequence alignment software TopHat2 [37], Clean Reads was compared with reference genes, and Mapped Reads was obtained, and were used for further analysis.

### Identification of differentially expressed genes (DEG)

Before differential gene expression analysis, we quantified the expression of genes, and FPKM (Fragments per Kilobase of transcript per Million fragments mapped) was used as an indicator of transcript or gene expression levels [38]. After normalizing genes expression levels, DEGs were obtained by comparison of the two transcriptome libraries using differential expression genes analysis software of DBSeq [39]. The Fold Change≥2 between two libraries was defined as the reference standard, with the Benjamini–Hochberg false discovery rate (FDR < 0.01) used to adjust the p-values.

### Functional annotation and enrichment analysis of DEG

Using the BLAST [40] software, the DEGs were compared with NCBI non-redundant protein (NR), Swiss-Prot protein database (Swiss-Prot), Gene Ontology (GO), Cluster of Orthologous Groups (COG), and Kyoto Encyclopedia of Genes and Genomes (KEGG) databases to obtain annotation information about the DEGs. And Gene Ontology (GO) including molecular functions, biological processes, and cellular components were obtained using the Blast2GO program (https://www.blast2go.com/) [41]. Pathway-enrichment analysis can further identify significantly enriched metabolic pathways or signal transduction pathways using the KEGG database and it was performed using the KOBAS software [42]. GO terms and KEGG pathways with Q values less than 0.05 were significantly enriched in DGEs.

### Real-time quantitative PCR (RT-qPCR)

In order to confirm the results of the DGE libraries, the specific primers of the 16 genes of interest were designed as listed in Table 1. Genes selected for RT-qPCR according to the DGE-tag copy number were also evaluated by the enrichment analysis of GO and KEGG pathways. RT-qPCR reactions were prepared with the TransStart^®^ Tip Green qPCR SuperMix (TRANSGEN BIOTECH), following the manufacturer’s instruction with 20 μL reactions (10 μL 2× SYBR Green Mix, 1 μL forward primers, 1 μL reverse primers, 1 μL cDNA, and 7 μL RNase-free H2O). Reactions were performed using the Bio-Rad CFX96TM Real-Time System (Bio-Rad, USA). Amplification conditions were as follows: initial denaturation at 95°C for 15 s, followed by 40 cycles of 95°C for 5s, and 58°C for 30 s for annealing. All of the samples were measured independently three times. Relative expression levels were calculated using the 2^−△△Ct^ method [43], the value stands for an n-fold difference relative to the calibrator. In this study, Bm18S rRNA gene was used as an internal reference gene.

**Table 1:** The primers used for real-time quantitative PCR analysis.

No.	Genes ID	Forward Primer	Reverse primer
1	BGIBMGA001613	CAGCCTCCAGACCCTAACAA	TGGTCATTGTCGAATCGGGA
2	BGIBMGA004810	TACCACCGGAGAAACAGCAT	ACCTCCACATCTTCAGCGAA
3	BGIBMGA005670	TTCAGAGAACGGTGGAAGCT	TCCGCTGTTTCAAAGCATCC
4	BGIBMGA007425	GAGAGGAGAGCTTCGGTACC	CAGGACTCCGAGGAACAGTT
5	BGIBMGA007517	GGGCCGAGGTTACAACTTTG	AACTGGGTCACGCATCACTA
6	BGIBMGA010846	ACATCACCGAATTGCTGACG	TCGTCTTCTTAGCGTCGTGA
7	BGIBMGA014115	AGCACAACCTGACTTCGGTA	TTTCCTTCCACACTTGCACG
8	BGIBMGA014116	AGCACAACCTGACTTCGGTA	CAAGGTACCAGCCAGAGGAA
9	BGIBMGA001075	ACGTACACCTGTAGCCCAAA	GGGTGAGTATTCCGCGACTA
10	BGIBMGA003969	GAACCACAGGATCTTTCGCC	CGCCTCAATGAGCTCAGTTC
11	BGIBMGA009686	ACAAGGATATATCGCCGCCA	GACGTGACAATTGATGCCGA
12	BGIBMGA011964	GGTGGACCTTGGTTATTGCC	GAGGTTCCTCGACTTCCACA
13	BGIBMGA013129	GGTCGGCTGGATAGATTGGA	TGATCCGCACTGAACCTCTT
14	BGIBMGA000388	GTTAGCGCCGATAAGCCAAA	GGGTAAGGACTCAACTGCCT
15	BGIBMGA002114	TCCTCGCTAAGCTGGATGAG	CCTCGGTGATGCTGTTGATG
16	BGIBMGA006878	TGACGCCGTTCAAATTGTGT	GCAAAGCAACTCCAACTCCA
17	Bm18S rRNA	CGATCCGCCGACGTTACTACA	GTCCGGGCCTGGTGAGATTT

## Results

### Morphological comparison of colleterial glands between H9 and P50

**Fig 1:**
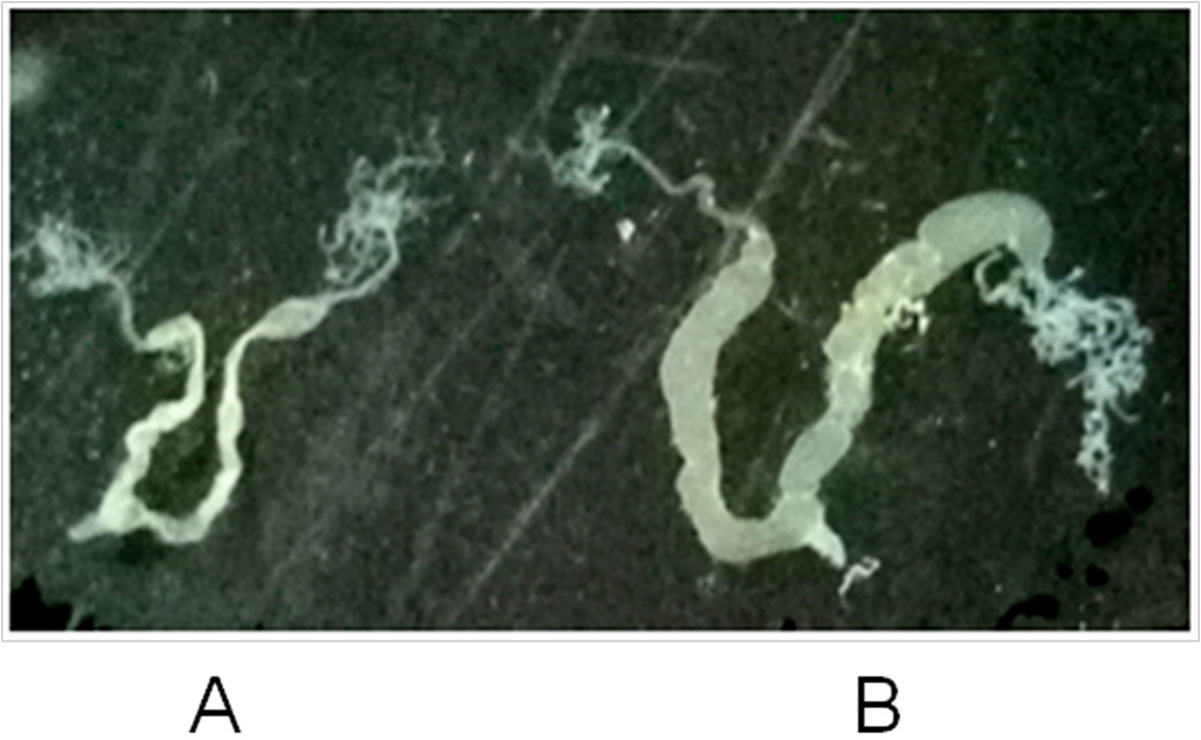
Morphological comparison of mucous glands. A: represent the colleterial glands of H9; B: represent the colleterial glands of P50. A colleterial glands including two part, the terminal part called dendritic branch and the stem part called storage.

In order to understand the morphological difference between H9 and P50 CG, we compared the intact tissues of the CG isolated from the virgin moths of the two strains (Fig 1). The CG are located on both sides of the oviduct, from the morphology, it can be divided into two parts: the terminal part called dendritic branch is responsible for mucus secretion, stem part is used to store the adehesive secretions, and both stem parts fuse in the vicinity of the base, and opening at the vaginal vestibule witch at the bottom of the fertilization tube [22]. The dendritic branch of P50 CG is more flourishing than that of H9, which suggested that the ability of secreting adehesive substance of P50 CG was stronger than that of H9. The stem part of P50 CG is bigger than that of H9, which suggested that the storage of adehesive secretions from P50 CG was more than that of H9.

### Transcriptome sequencing and statistics of gene expression

**Fig 2:**
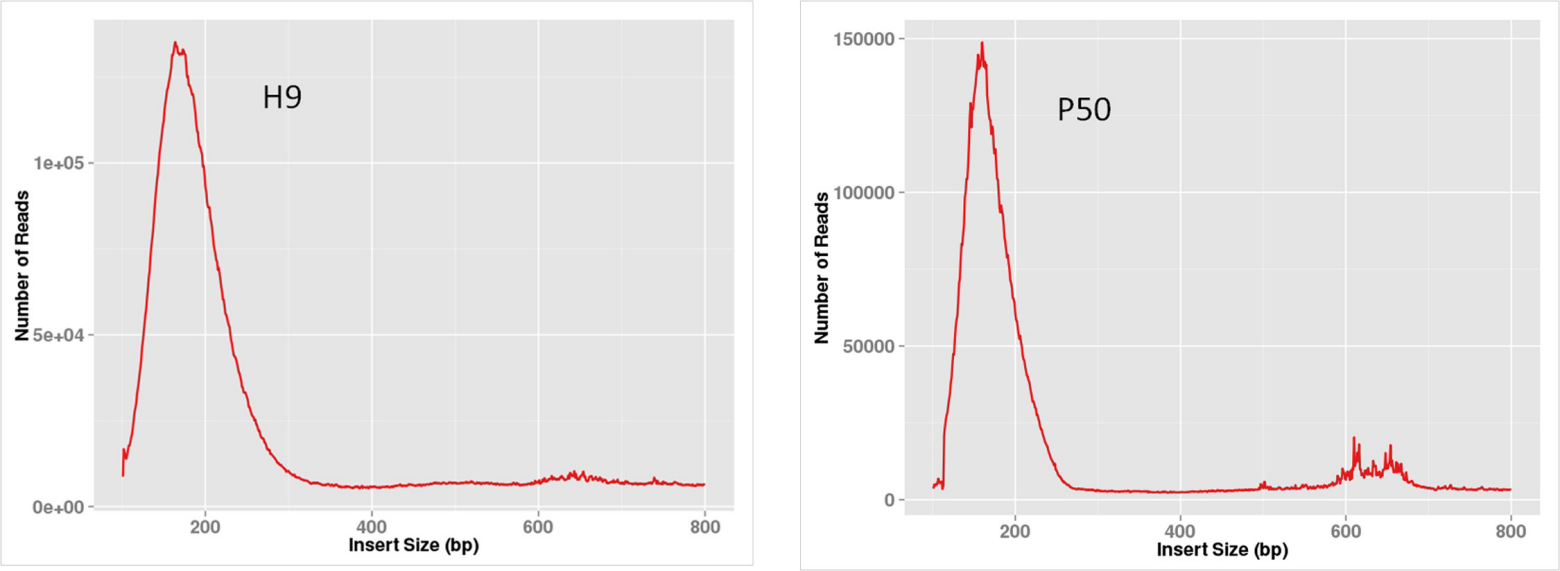
Insertion length analog distribution map. The X-axis is the distance between the start and stop points of double ended reads on the reference genome, the range from 0 to 800bp; the Y-axis is the number of inserted segments at different distances between the start and stop points. The main peak of the graph falls near 180 bp and the peak shape is narrower.

Illumina DGE analysis was performed to obtain the overview of the silkworm transcriptome in different samples. The DEGs between H9 and P50 strains were searched and analyzed according Illumina DGE analysis in this study. A total of two cDNA libraries (H9 and P50) were sequenced, the results of the two libraries showed that 19,896,957 and 20,446,366 sequence reads were generated after removing the adaptors and low quality sequences (Table 2). The GC content of two libraries in each library is about 45%, and the cycle Q30% is greater than 89.05%. Therefore, the sequencing data were enough for further analysis with the quality and accuracy. Most of the reading conforms to the position of the silkworm genome. All the unigenes matched previously described sequences of more than 45% coverage. The insertion length distribution of unigenes has a similar pattern in two libraries, indicating that there was little bias in the construction of the two cDNA libraries (Fig 2). These reads were submitted to SRA at NCBI under the accession no. SRX3973905 (P50) and SRX3973906 (H9).

**Table 2:** Distribution of tags from H9 and P50 library.

Items	H9	P50
Total Reads (pair-end)	19,896,957	20,446,366
GC Content (%)	44.46	44.74
%≥Q30 (%)	90.83	89.05
Mapped Reads (single-ended)	27,275,078	20,089,155
Mapped Ratio (%)	68.54	49.13
Uniq Mapped Read (single-ended)	26,139,507	18,652,579
Uniq Mapped Ratio (%)	65.69	45.61

**Fig 3:**
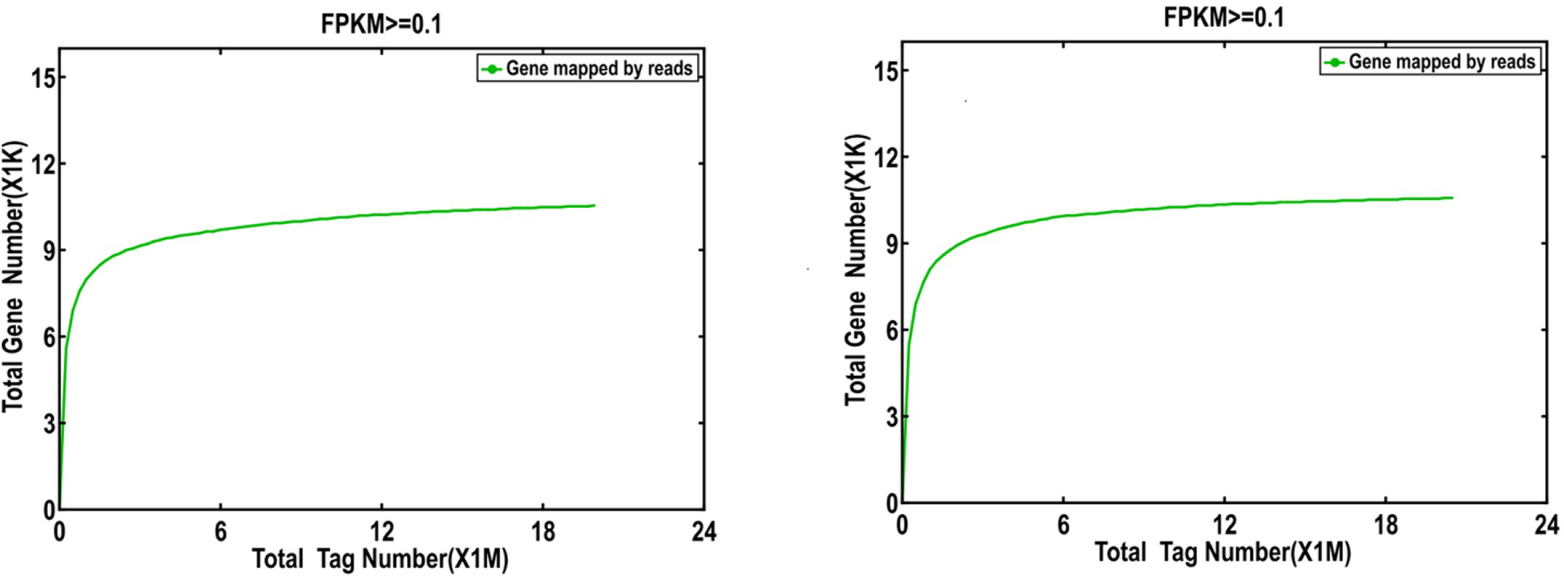
Saturation map of sequence data. The saturation curve is plotted by dividing the Mapped Reads equally into 100 and increasing the number of genes examined to see the number of detected genes. The X-axis is the number of mapped reads (in 10^6^ units), and the Y-axis is the number of genes detected (in 10^3^ units). Genes that express FPKM ≥ 0.1 are defined as expressed genes.

Sequencing saturation was analyzed to estimate whether or not the sequencing depth was sufficient for transcriptome coverage. The results showed that when the total tag number reached more than 2 million, the number of the detected genes was almost saturated (Fig 3). We reached the sequencing depths of each DGE approximately 4.0 million in each library, which meet the requirement for further experiment. The above results confirm that the two DGE libraries are reliable.

### Identification of differentially expressed genes

**Fig 4:**
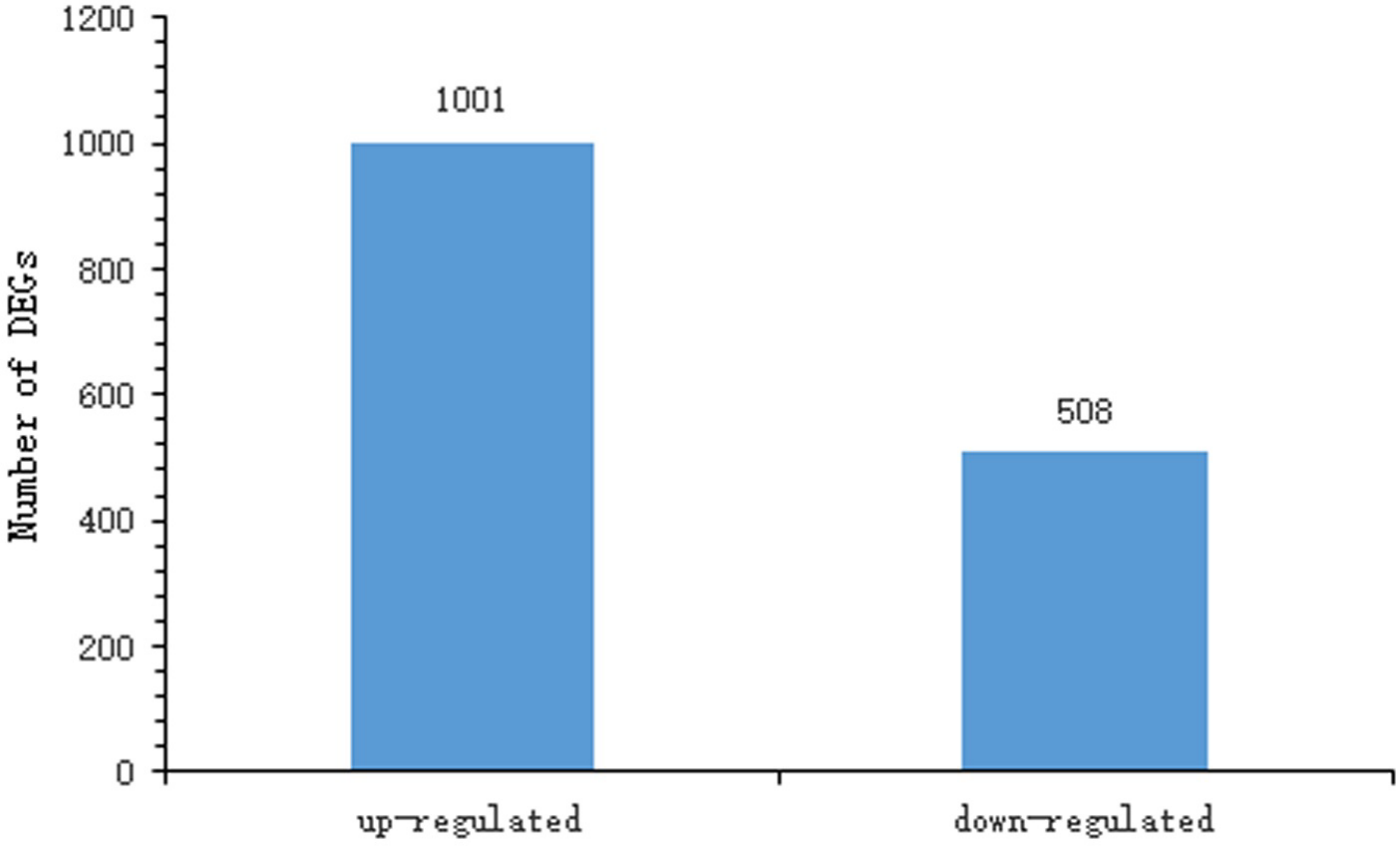
The differentially expressed unigene sequences obtained from H9 and P50 library. Among the 1,509 genes, 1,001 are up-regulated and 508 are down-regulated in P50 individuals compared with H9 individuals.

**Fig 5:**
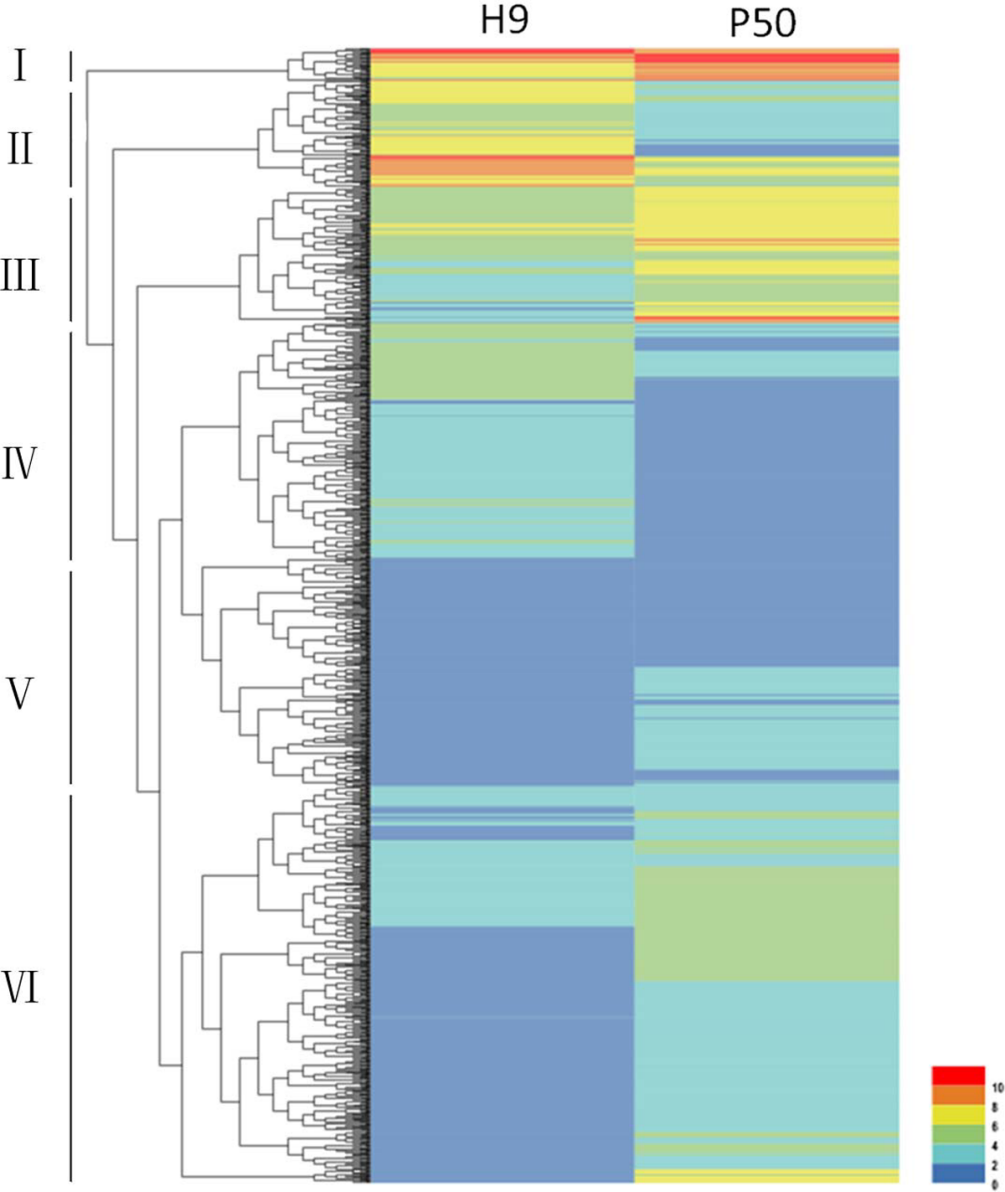
Cluster analysis of differential gene expression pattern. Column indicates H9 and P50 strains, row represent a gene. Expression levels are shown in different colors (log_2_FPKM + 1). Based on the expression pattern, genes could be divided into two clusters. The group II and IV contained genes expression levels were higher in H9 than that P50. The genes in group I, III, V and VI were expressed highly in P50 than that of H9.

After normalizing genes expression levels, FDR<0.01 and Fold Change≥2 were used as the threshold to evaluate the significance of DGE between the two samples. A total of 1,509 provided a BLAST result in 1,599 unigenes (S1 Table), of which 1,001 unigene were up-regulated and 508 were down-regulated (Fig 4). S2 Table shows the species that best matches for each unigene. Most of the annotated sequences had the highest homology with sequences of *Bombyx mori* (88%) and *Danaus plexippus* (8%). Due to similar gene expression pattern usually mean functional correlation (33). In order to observe the pattern of overall gene expression, we conducted hierarchical clustering analysis, which compared expression of genes in the two libraries, based on the sample's log_2_FPKM + 1. These genes are divided into two clusters (Fig 5). Genes expressed different in the two clusters sorted into six groups. The genes in group II and IV were expressed highly in H9 than that in P50. But in group I, III, V and VI the results were conversely, the genes expression levels were higher in P50 than that H9. These results revealed that the gene expression differences between P50 and H9 CG.

### Functional annotation and enrichment analysis of DEG

**Fig 6:**
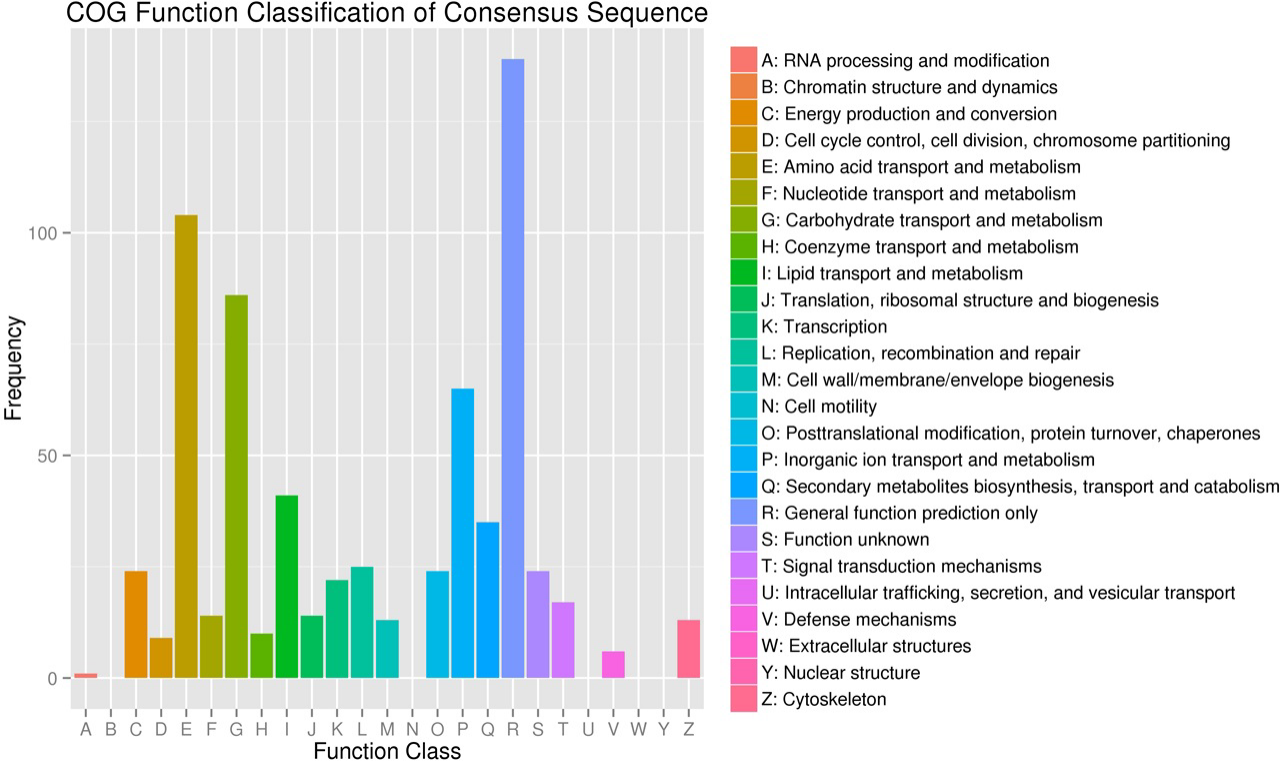
COG classification of annotated differentially expressed genes. Detected genes were categoried (25 catagories from A to Z). X-axis represents function class and Y-axis represents the number of genes.

**Fig 7:**
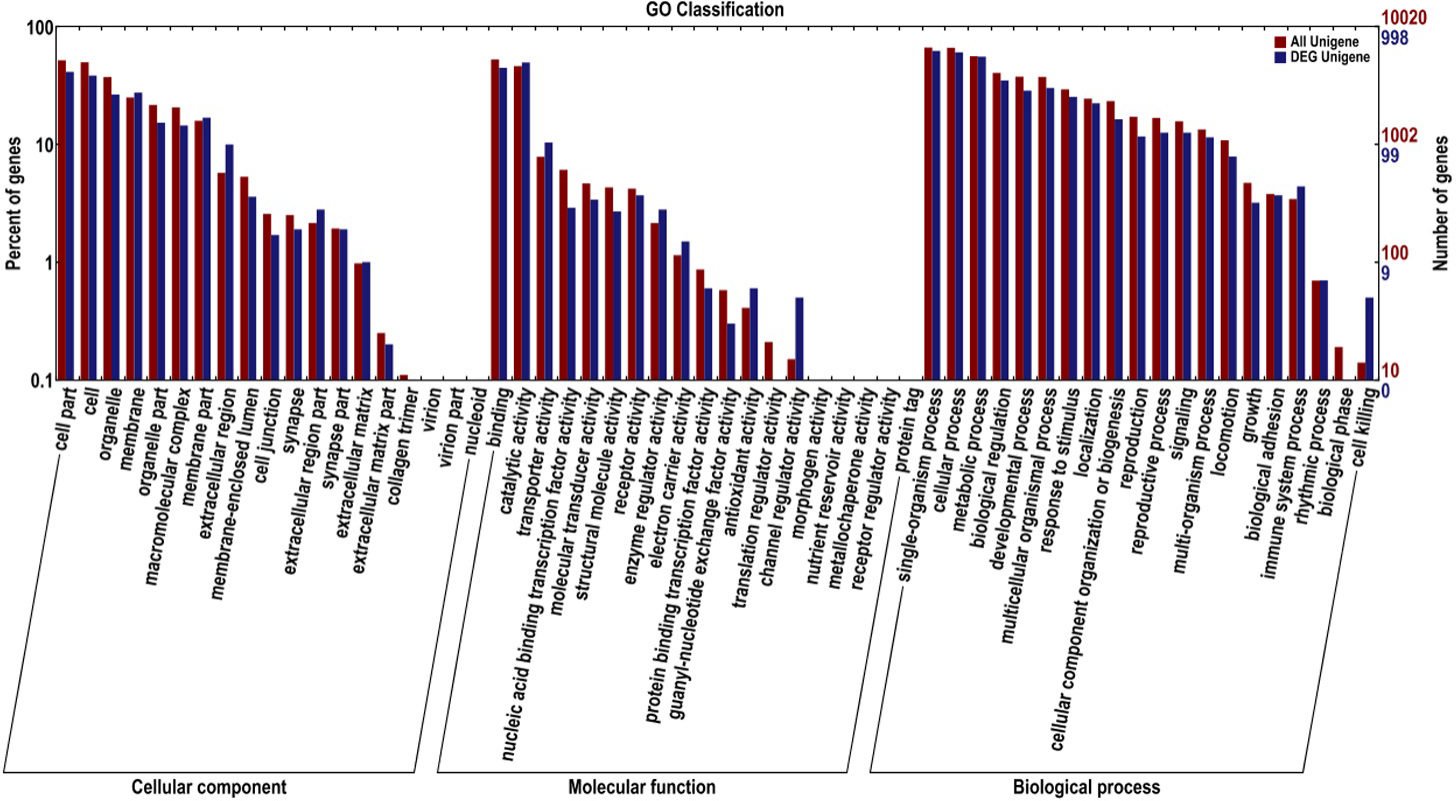
GO classification of annotated differentially expressed genes. The DEGs were categorized into 58 functional groups and were summarized in three main categories: biological process, cellular component and molecular function. The y-axis on the left indicates the percentage of the number of genes in that main category, the y-axis on the right indicates the number of genes in category.

To understand the function of the differentially expressed genes, all of the 1,509 DEGs were mapped against different data bases (Table 3). A total of 453 genes had at least one COG classification (Fig 6). Among the 25 categories, “General function prediction only” was the largest group (139, 30.7%). Followed by “Amino acid transport and metabolism” (104, 23.0%), “Carbohydrate transport and metabolism” (87, 19.2%), “Inorganic ion transport and metabolism” (65, 14.3%), “Lipid transport and metabolism” (41, 9.0%). Of the DEGs 998 unigene sequences best aligned against GO data base, and categorized into 58 functional groups (Fig 7). This map shows the gene enrichment of the secondary function of GO under the two backgrounds: differentially expressed gene background and whole genome background, and the secondary function with marked proportion difference indicated that the differentially expressed genes were different from the whole genome. Among three ontologies (molecular function, cellular component and biological process) of the GO classification, genes related to “nucleic acid binding transcription factor activity”, “guanyl-nucleotide exchange factor activity”, “translation regulator activity”, “channel regulate activity”, “biological phase”, and “cell killing” were predominantly. These data will provide a valuable resource for investigating the possible function of the CG in the silkworm.

**Table 3:** Differences in the number of expression genes.

DEGs Set	Annotated	COG	GO	KEGG	Swiss-Prot	nr
H9_vs_P50	1,509	453	998	359	976	1,509

**Fig 8:**
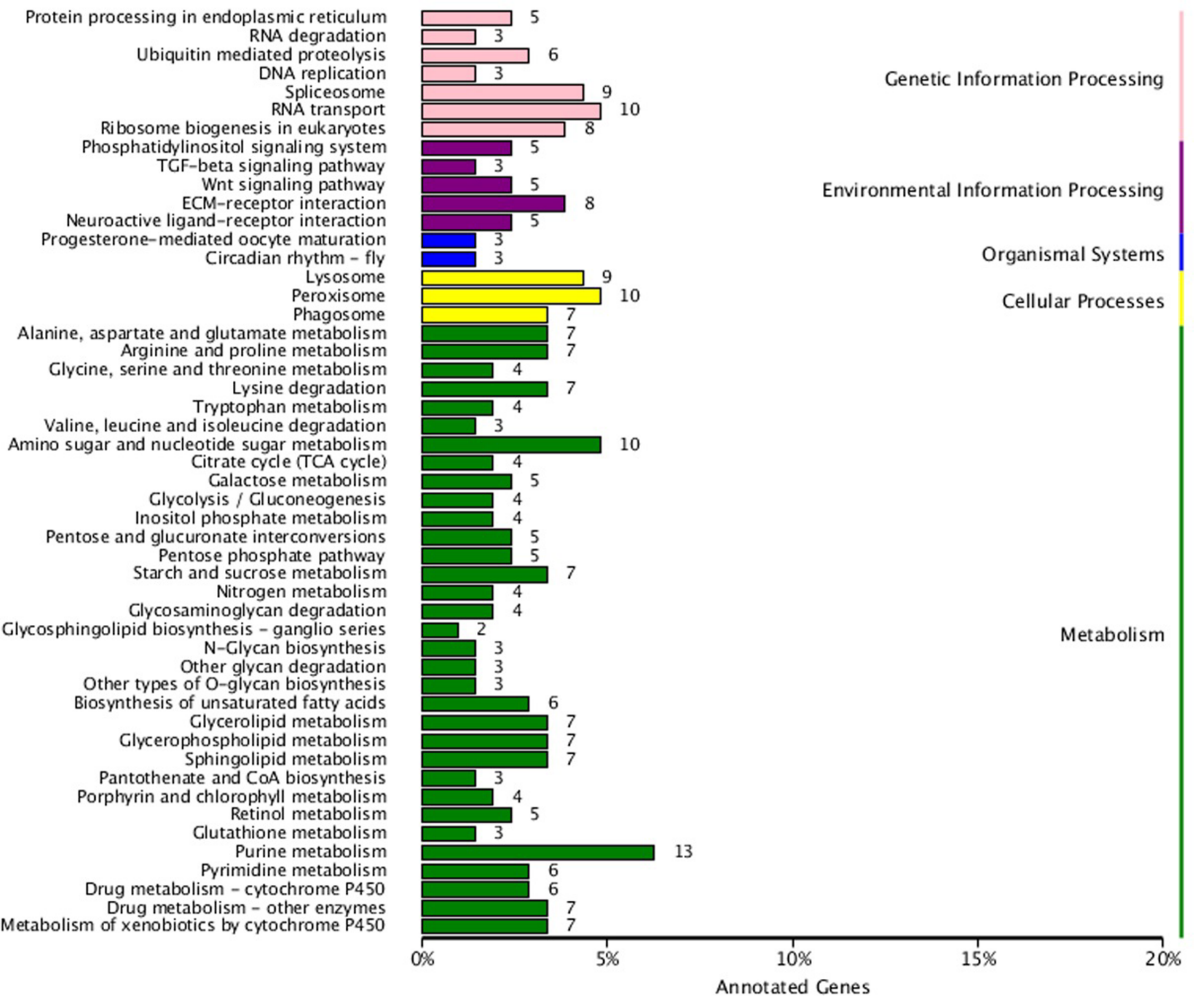
KEGG classification map of differentially expressed genes. The y-axis shows the KEGG metabolic pathway, and x-axis represent the number of genes annotated to the pathway and the proporation of the total number of annotation. Different colors present different metabolic functions.

**Fig 9:**
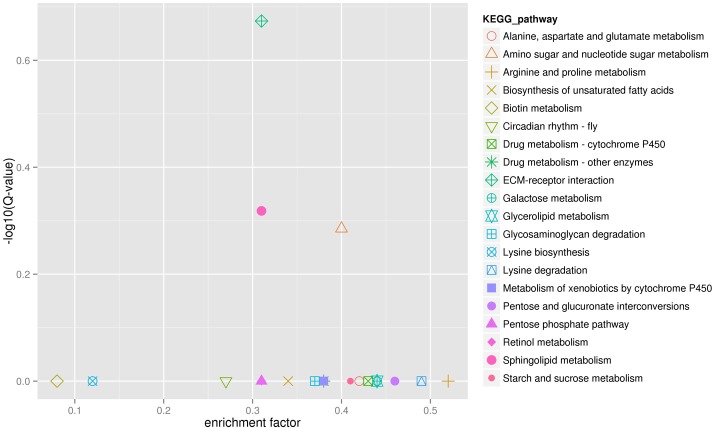
KEGG enrichment scatter plot map of differentially expressed genes. Each graphics in the diagram represents a KEGG pathway, and the pathway name is shown on the right of legend. The x-axis is enrichment factor. The y-axis is log10 (Q value), and the Q value is the *P* value after the multiple hypothesis test.

To investigate the biochemical pathways, the DEGs were aligned against the KEGG database. And, the results were compared with the entire transcriptome background. In total, 359 of the 1,509 DEGs shaw the KEGG pathway ID (KO ID), which can be categorized into 104 pathways (Fig 8; S3 Table). Most pathways in the KEGG classification were involved in metabolism. In order to analyze differential expressed genes are present on one pathway (over-presentation) or not, the pathway enrichment were analyzed of differential expression genes (Fig 9), and the first 20 paths with significant Q values in the smallest are shown in (Fig 9). Enrichment factors and the enrichment levels of differential genes were inversely proportional in the pathway, and the reliable of enrichment significance was directly proportion to the numerical of Y-axis. Of these pathways, ECM-receptor interaction, sphingolipid metabolism, and amino sugar and nucleotide sugar metabolism pathway were most significantly enriched, which including 25 genes may be associated with the CG’ development and mucus secretion.

### RT-qPCR validation of differentially expressed genes

**Fig 10:**
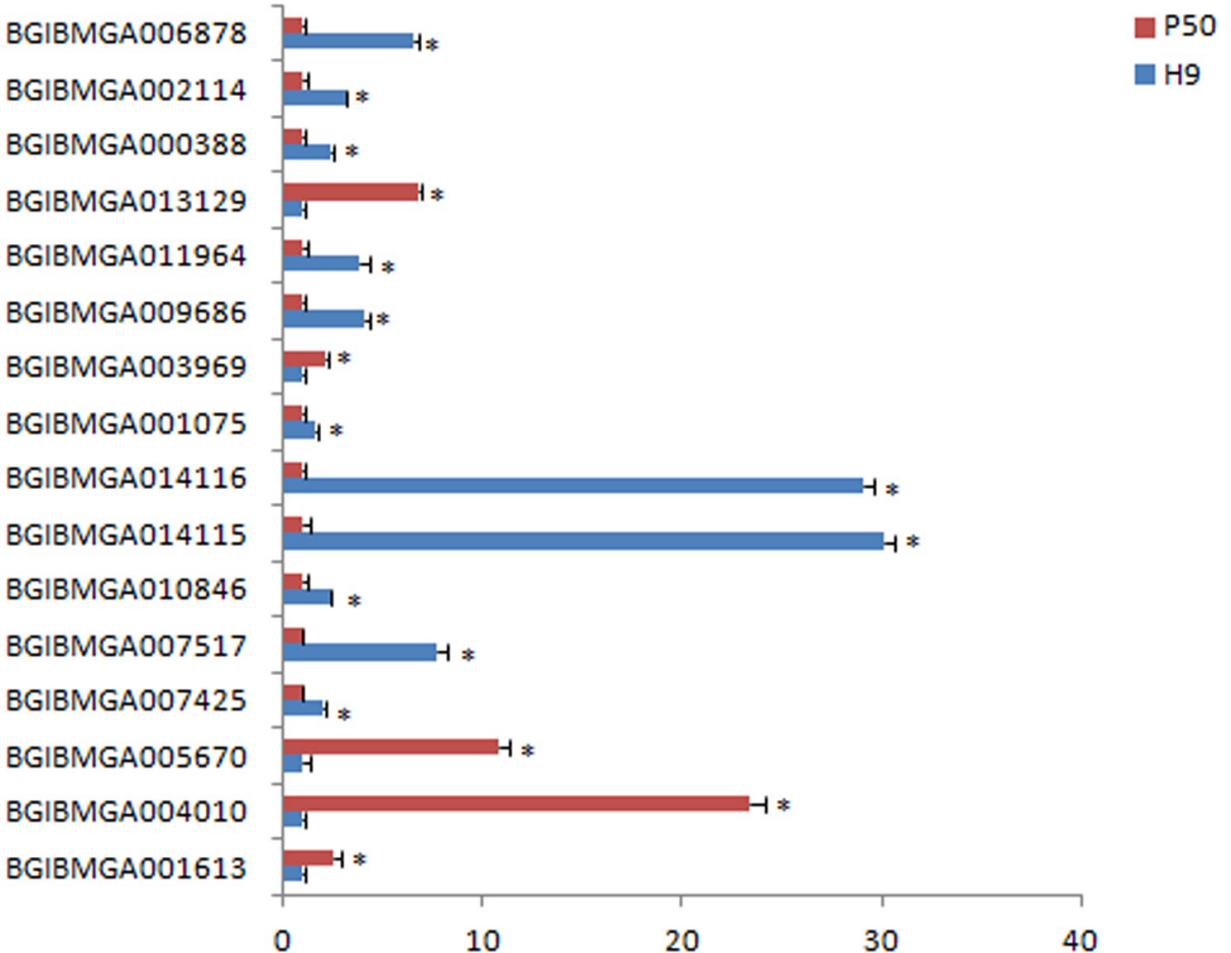
RT-qPCR analysis of 16 DEGs in the colleterial glands of H9 and P50 individuals of silkworm. The x-axis shows relative expression levels (blue colour represents the H9, and red colour represents P50).

To validate the reliability of the transcriptome sequencing, a set of 16 genes were selected for RT-qPCR analysis. Five of the 16 genes (BGIBMGA001613, BGIBMGA004010, BGIBMGA005670, BGIBMGA003969, and BGIBMGA013129) were up-regulated and remaining were down-regulated (Fig 10), like the transcriptome sequencing, although the relative expression level varied. This suggested a strong positive correlation between RT-qPCR and transcriptome data.

## Discussion

Despite a series of studies have made significant progress in morphology and composition of CG glue, the molecular mechanism of rapid development of CG and excessive mucus secretion is unclear. In this study, Illumina SBS sequencing technology was as a high-throughput DNA sequencing method analyzes differentially expressed genes in the libraries prepared from the CG isolated from H9 and P50 strains. The results obtained were used to understand the mechanism of the development and secretory function of CG.

In the present study, 1509 differentially expressed genes with 1001 up-regulated and 508 down-regulated in P50 CG compared with H9. We observed that the relative expression of most genes in DGEs was higher in P50 than in H9. Thus, we speculate that these highly expressed genes in P50 may be related to the molecular mechanism of the CG’s rapid development and mucus secretion. Of course, the high expression genes in H9 may be involved in the regulation of this molecular mechanism, and lead the CG to reduce mucus secretion.

The GO classification analysis showed that the sugar transmembrane transporter activity in molecular function was significantly enriched, such as (BGIBMGA006529, BGIBMGA0010728, BGIBMGA0010722, BGIBMGA004508, BGIBMGA004510, BGIBMGA005424, and BGIBMGA005603) genes were related to facilitated trehalose transporter. What’s more they were more significantly expressed in P50 than in H9. The result is same with the study: The activity of trehalose, which is used as energy substrates in insect, is closely related to the rate of protein synthesis in CG, what’s more the changes of the two are consistent [44]. Furthermore three sugar transporter proteins (BGIBMGA004525, BGIBMGA004527, and BGIBMGA000223) were identified and highly expressed in P50 compared to H9.

The KEGG analysis results demonstrated that the ECM-receptor interaction pathways, sphingolipid metabolism pathways, and amino sugar and nucleotide sugar metabolism pathways were significantly enriched, which indicated that the development of CG and the secrete of glue substance were related to the metabolism of sphingolipid, amino sugar and nucleotide sugar, and the interaction of ECM-receptor. Further the ECM-receptor interaction pathways synaptic vesicle glycoprotein (BGIBMGA001498, BGIBMGA002430) was significantly expressed in P50 than H9, and it is a specific protein located on the membrane of synaptic vesicles and plays an important role in neurotransmitter release [45]. Amornsak *et al*. found that water (85%) and protein (11%) are the main components of CG. One of the biggest protein’s molecular weight is about 240 kDa [14]. So we supposed that protein molecules are glycosylated, which provides new insights into the chemistry of secretions. The enrichment sphingolipid metabolism pathways suggested that neural regulation may be involved in the development of the CG. Further, the rapid development of CG and mucus secretion requires a large number of ATP produced by carbohydrate metabolism.

RT-qPCR analysis suggested a strong positive correlation with transcriptome data. Therefore, the transcriptome data were satisfied for further analysis. And RT-qPCR analysis showed that the N-acetylneuraminate lyase-like gene’s (BGIBMGA004810) expression was 23 times higher in P50 strain compared to H9. It is generally believed that the main function of the enzyme is to provide nutrition, as the degradation of free sialic acid produces carbon and energy sources for the microorganism [46]. The galactokinase-like gene (BGIBMGA005670) expression was relatively 11 times greater in P50 than that of H9; what’s more Thoden *et al*. found that galactokinase plays a key role in normal galactose metabolism by catalyzing the ATP-dependent phosphorylation of α-D-galactose to galactose 1-phosphate [47]. The synthesis of the protein requires energy substrates metabolism to provide a lot of energy. So, these genes may be involved in the fundamental process for the development and viscous secretions of CG.

In the down-regulated genes, the expression differences of beta-N-acetylglucosaminidase isoform A (BGIBMGA014115) and beta-N-acetylglucosaminidase 2 (BGIBMGA014115) in P50 and H9 were relatively large and the expression level in H9 was 29 times higher than in P50. Okada *et al*. found that beta-N-Acetylglucosaminidase is a major glycosidase involved in glycoconjugate degradation. Therefore, we hypothesize that the components of secretions in H9 are degraded and result in changes of their contents and viscosities. Especially would affect the chemical properties of secretions.

To detect the conservation of these differentially expressed genes between H9 and P50 individuals, we also observed the statistical distribution of 1509 differentially expressed genes in the two libraries. Of the DEGs, 88% unigene sequences were annotated to *Bombyx mori* and 8% were annotated to *Danaus plexippus*, indicating the reliability of data. Further silkworm sequences are highly homologous to other species sequence, particularly with insects. However, further investigation is necessary to identify the new genes in *Bombyx mori*. In addition, 90 differentially expressed genes with unknown functions were observed, and these genes may be involved in the regulation of other important genes and may be crucial to distinguish between these functions.

In conclusion, we obtained 1509 differentially expressed genes between H9 and P50 individuals through analysis. The current results not only provide important clues for future research, but also extend our understanding of the synergistic effect of genes in the development of CG and other secretory.

## Supporting information

S1 Table H9_vs_P50.annotation. (XLS)

S2 Table H9_vs_P50.nr. Annotation. (XLSX)

S3 Table H9_vs_P50.KEGG. (XLS)
